# Influence of Oxygen Content in the Protective Gas on Pitting Corrosion Resistance of a 316L Stainless Steel Weld Joint

**DOI:** 10.3390/ma16175968

**Published:** 2023-08-31

**Authors:** Mohammad Maroufkhani, Soroosh Hakimian, Alireza Khodabandeh, Iulian Radu, Lucas A. Hof, Mohammad Jahazi

**Affiliations:** 1 Department of Mechanical Engineering, École de Technologie Supérieure, Montreal, QC H3C 1K3, Canada; soroosh.hakimian.1@ens.etsmtl.ca (S.H.); alireza.khodabandeh@etsmtl.ca (A.K.); lucas.hof@etsmtl.ca (L.A.H.); 2 PCL Industrial Constructors Inc., Edmonton, AB T6E 3P4, Canada; iradu@pcl.com

**Keywords:** 316L SS, pitting corrosion, oil and gas pipeline, discoloration, oxygen concentration

## Abstract

Gas tungsten arc welding (GTAW) is commonly used for joining pipelines; however, it often leads to discoloration in the heat-affected zone (HAZ). In this study, 316L pipes were welded with different concentrations of oxygen present in the argon purge gas during welding. The objective of this study was to investigate the effect of oxygen concentration in the protective gas on the pitting corrosion resistance of welded pipes. The experimental results showed that the thickness of the oxide layer formed in the HAZ depends on the concentration of oxygen in the protective gas. Increasing the oxygen concentration in the protective gas resulted in an increase in pitting corrosion resistance until a critical value, beyond which the resistance decreased. The results showed that the thickness of the oxide layer formed in the HAZ depends on the concentration of oxygen in the protective gas. Increasing the oxygen concentration in the protective gas increased the pitting corrosion resistance until a critical value, beyond which the resistance decreased due to the formation of iron oxide. This study provides valuable insights for improving the corrosion resistance of welded pipes in the oil and gas industry.

## 1. Introduction

Although stainless steels (SS) are known for their good corrosion resistance, some industrial welded joints made of SS are prone to pitting corrosion. The presence of chloride ions can break down the passive film, leading to the formation of pits in both welded and non-welded SS. Pitting corrosion is considered one of the most dangerous types of corrosion due to its ability to cause localized damage and lead to sudden failures in equipment or structures [1,2].

The welding process is a complex and intricate process that requires careful attention to various parameters. These parameters have a significant impact on the quality and performance of welded joints. Among the critical parameters that need to be monitored and controlled are voltage, current, welding speed, shielding, and backing gas, as well as pre-and post-weld heat treatments [3,4]. Therefore, these parameters must be carefully monitored and adjusted to ensure the production of high-quality welded joints that meet the required performance standards [5,6]. To this end, during welding, particularly for the root pass, it is necessary to remove air from the fusion zone by employing an inert purging gas on the back side of the weld (i.e., from the inside of the tube). It has been reported that the presence of oxygen at atmospheric concentration levels, i.e., about 210,000 ppm, can lead to the formation of an oxide layer on the surface of the weld root [7]. Some authors have reported that this oxide layer can lead to contamination that results in reduced mechanical properties and lower corrosion resistance in the weldments. Specifically, the occurrence of pitting corrosion in the HAZ of welded austenitic SS joints has been reported by many authors [7,8,9]. To reduce and alleviate the susceptibility to pitting corrosion of welded joints, Argon (Ar) has been used as a purging gas in the GTAW of various austenitic SS. Although pure Ar would be the ideal option, its rising cost provides a strong incentive to switch to a lower-purity option [7,10].

The typically studied corrosion parameters include the passive current density (i_passive_), passive potential (E_passive_), pitting current density (i_pit_), and pitting potential (E_pit_). The passive current density is defined as the current density required to maintain a passive film on the metal surface, the E_passive_ value indicates the passive potential for a given metal, which is the potential at which the metal surface becomes passive, causing a protective oxide film to form, and the i_pit_ and E_pit_ values indicate the current density required to initiate localized corrosion in the form of pits on the metal surface and the potential at which the metal surface begins to undergo localized corrosion in the form of pits, respectively [11].

Ling et al. studied the oxidation behavior of 304L during GTAW and found that, without inert gas shielding, the surface near the weld oxidized at a higher rate [12]. Specifically, they observed that the extent of oxidation increased as the distance to the fusion line decreased, leading to the growth of an oxide film and cracks on the oxide film. The HAZ region mainly consisted of the Fe-Cr oxides and the oxidation extent and severity increased with the welding temperature [12]. Huang et al. found an outer layer of Cr_2_O_3_ and Fe_2_O_3_ and an inner layer of FeCr_2_O_4_ in samples exposed at 400 °C, while at 600 °C, an oxide film with a Fe-rich outer layer and a Cr-rich inner layer in 316L SS was observed [13]. According to Xie et al., Fe and Cr oxide were detected in the oxide scale of 316L SS, while the high vacuum-generated initial oxide layer consisted of a Fe_3_O_4_ outer layer and an inner layer enriched with both Fe and Cr [14].

In the discoloration area of the weld zone, a range of colors from straw yellow to blue, purple, or even black is observed. The extent of colors is influenced by a combination of factors, including oxidation and temperature changes during welding [15]. One of the primary factors contributing to the colors observed in the HAZ is the formation of oxide layers on the metal surface [15]. The thickness and composition of the oxide layer can vary depending on factors such as the welding process, the atmosphere, the heat input, the exposure time, and the specific metal being welded [15]. These oxide layers interact with light, resulting in the selective absorption, reflection, and scattering of different wavelengths, and leading to the observed colors [16]. Furthermore, the thickness of the oxide layer also influences the observed color. For example, it has been reported that thicker oxide layers can result in a broader range of colors, including blues, purples, and even black, while thinner oxide layers appear as straw yellow or light shades [17].

Equation (1) illustrates the Ray optic concept, where an incident light ray (*I*) interacts with the surface of an oxide layer. The incident ray splits into a reflected ray (*R*1) and a transmitted ray (*T*1) that passes through the oxide layer. Upon reaching the oxide layer/bulk material interface, the transmitted ray is reflected (*RT*1) and travels back through the oxide layer before exiting into the air. The color that is observed depends on the optical path difference (*OPD*) between the reflected ray (*R*1) and the transmitted-reflected ray (*T* *RT*1). This path difference is determined by the refraction angle, which follows Snell’s law (*θ*2), the thickness of the oxide layer (*t*), the refractive index of the oxide layer (*n*2), and the wavelength of light (*λ*). The refractive index (*n*2) is wavelength-dependent, influencing the interference pattern [16,17,18].

OPD = 2·(*n*2(*λ*)·*t*·*cos*(*θ*2)) (1)

In the case of oxide films, which could be composed by different layers of materials with different refractive indices, interference effects can occur, as the thickness of the thin film influences the optical path difference [16,17]. The optical path difference determines the phase relationship between the interfering waves, which, in turn, affects the resulting observed color. Depending on the film’s thickness and the wavelength of the incident light, different colors will be determined [17].

The current study aims to fill existing gaps in the literature by investigating the impact of oxygen content in the purging gas during GTAW on the discoloration levels of the root joint area and the pitting corrosion resistance of AISI 316L SS. Specifically, this research focuses on evaluating the pitting corrosion resistance of steel without removing the discoloration. The present study aims to investigate the critical value of oxygen impurity in argon backing gas, with a specific focus on its influence on the pitting corrosion resistance. By identifying the optimum oxygen content for enhanced corrosion resistance, this research contributes to the development of practical solutions for industries that are seeking both effective welding outcomes and cost efficiency. The principal objective of this research is to quantify the impact of variations in oxygen content on the pitting corrosion resistance of the welded joint. Therefore, four different oxygen contents in backing gas were selected for the analysis. A combination of microscopic and chemical analysis methods, as well as thermodynamic modeling, were used to characterize the discoloration, then pitting corrosion tests were carried out and the oxide layers’ nature and compositions were analyzed. Then, the operating mechanisms are discussed. Finally, recommendations on operational conditions are proposed.

## 2. Materials and Methods

The specimens used in this work were made of 316L SS with a chemical composition (wt.%) as indicated in Table 1. The chemical composition was determined through the utilization of spectrometry, a technique well-regarded for its precision in analyzing material composition. The analysis process involved taking multiple measurements to ensure accuracy and repeatability. In fact, the measurements were carried out five times to establish the reliability of the results. To perform the chemical composition determination, a Thermo Scientific ARLTM 4460 Optical Emission Spectrometer (Thermo Fisher Scientific Inc., Waltham, MA, USA) was employed. In this study, 316L SS specimens were welded using GTAW with varying concentrations of oxygen in the purging gas. The effect of oxygen content on the discoloration levels and pitting corrosion resistance of the root welded joints was investigated. The interior of the pipe was purged with argon gas containing specific oxygen concentrations, ranging from 50 to 5000 ppm. The thermodynamic software FactSage was used to predict the formation of possible phases based on oxygen content and temperature. Potentiodynamic anodic polarization tests were conducted to determine the pitting potential. Discoloration images were acquired using a 3D scanner, and the oxide layer was analyzed using scanning electron microscopy (SEM) with the help of mounting techniques and ion milling methods. The detailed methodology and analysis procedures are described in the subsequent paragraphs.

**Figure 1:**
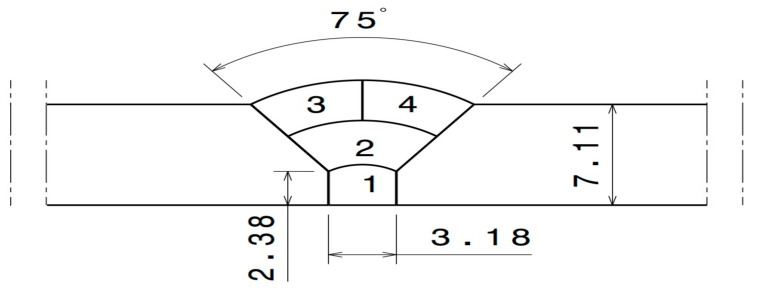
Weld zone geometry (dimensions in mm).

The pipe diameter was 154.4 mm (6 in) and the thickness of the base metal was 7.11 mm (0.285 in). GTAW was used for welding the 316L SS pipe. A total of four (4) passes were applied for welding to be as close as possible to the actual industrial conditions. The joint geometry is shown in Figure 1. The welding parameters of each of the four welding passes shown in Figure 1 are presented in Table 2. Before welding, the interior of the pipe (i.e., the back of the weld) was purged with Ar gas containing a specific oxygen content. Four different oxygen concentrations, namely 50, 200, 500, and 5000 ppm, were used for the purging, as indicated in Table 3. The oxygen gas used for welding was obtained from commercially available sources offering varying degrees of argon purity. To ensure its suitability, the gas underwent meticulous analysis conducted by Airliquid, a distinguished company specializing in precise gas assessments. Throughout the welding procedure, continuous monitoring of oxygen concentration was undertaken using the PurgEye 300 instrument. The initiation and maintenance of gas purging was meticulously executed until the desired and predetermined oxygen level was successfully attained. To create an environment conducive to accurate measurements, the pipe was effectively sealed from both ends. One end of the pipe was subjected to inert gas introduction, while, at the opposite end, the oxygen concentration was carefully gauged. The formation of possible phases as a function of the oxygen content and temperature was predicted using the thermodynamic software FactSage [19].

FactSage is a comprehensive software package widely used in the field of computational thermochemistry. Originally designed for pyrometallurgical processing, FactSage has evolved to encompass a wide range of applications. The Equilib module within FactSage performs Gibbs energy minimization calculations, enabling the determination of chemical species concentrations in systems reaching a state of chemical equilibrium. Additionally, the phase diagram module offers the capability to calculate various types of phase diagram sections, providing insights into phase transformations, stability ranges, and boundaries of different phases. In this study, the Equilib and Phase Diagram modules of FactSage were utilized to analyze the thermodynamic behavior of the steel [19].

Potentiodynamic anodic polarization (PAP) was used for determining the pitting potential of the HAZ. The tests were performed based on the ASTM G5-13 standard [20]. The size of the test specimens was 15 × 15 mm., with an area of 1 cm² that was exposed to the solution and examined. Each sample was placed in a flask containing the test solution, 0.5 M NaCl and 0.5 M H_2_SO_4_, for the pitting corrosion tests. The open circuit potential (OCP) values, also referred to as the corrosion potential, for each experiment was measured with a delay of 300 s.The polarization curve was plotted from 50 mV below OCP to 2000 mV (vs. Ag/AgCl) at a potential rate of 0.0016 Vs^−1^, as suggested by the literature [21]. Potentiodynamic polarization tests were repeated three times to determine the average pitting potential (i_pit_) for each condition. The tests were performed at 23 ± 2 °C according to the ASTM G5-13 standard test method.

For the electrochemical polarization tests, an electrochemical cell consisting of three electrodes was deployed. The 316L sample (working electrode (WE)) and the platinum counter electrode (CE) were immersed in the electrolyte and connected to a saturated Ag/AgCl reference electrode (RE) by a salt bridge. The sample holder used for holding the 316L sample (WE) was provided by Redox Company, (Norrköping, Sweden) and the electrochemical cell, CE, and RE were obtained from Pine Research Inc. The electrodes were connected to an Autolab potential/galvanostat PGSTAT302N potentiostat (Metrohm, FL, USA), which was controlled by a computer using ANOVA software version 2.1.6 (Metrohm Autolab B.V, Utrecht, The Netherlands).

In order to determine the pitting parameters (E_pit_, i_pit_, i_passive_, E_passive_) in this study, the inflection point method was employed, as suggested by the literature [22,23,24]. The method suggested by researchers involves identifying the point on the polarization curve where a change in slope occurs. It is characterized by the intersection of two tangent lines that represent the transition from activity to passivity (E_passive_, i_passive_) and from passivity to pitting (E_pit_, i_pit_).

**Figure 2:**
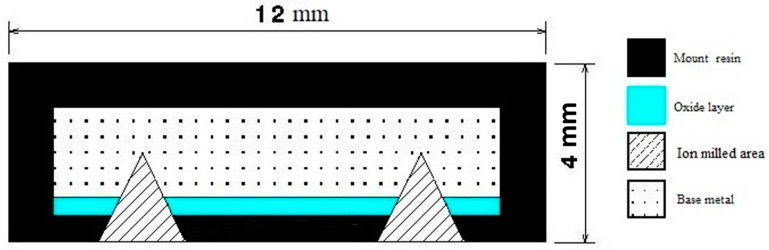
Schematic showing the ion mill regions of the samples for accurate determination of the thickness of the oxide layers and their composition.

Discoloration images were acquired using a Keyence VR2500 3D scanner (KEYENCE CANADA INC, Mississauga, ON L5N 0A4, Canada) to examine the oxide layer. Subsequently, the oxide layer was carefully analyzed using scanning electron microscopy (SEM). Two high-resolution SEM instruments, the Hitachi SEM TM3000 and the Hitachi SU8230 FE-SEM (Hitachi High-Tech Canada, Toronto, ON, Canada), were employed for the analysis. To ensure the preservation of the delicate oxide layers, a combination of mounting techniques and cross-section and face ion milling methods were employed. Figure 2 illustrates the specific technique that was developed and employed in this investigation. This developed method involved the use of hot mounting resin and cross-section and surface ion milling, effectively protecting the integrity of the oxide layers. For the cross-section part, ion milling was conducted for 2 h at 6 kV with a speed of 60 reciprocations per minute and a swing of ±15 degrees. This was followed by 1 h of ion milling at 5 kV with a speed of 30 reciprocations per minute and a swing of ±30 degrees. For the surface ion milling, a 3 min process was carried out at 5 kV with 15 reciprocations per minute and a swing of ±60 degrees. In order to prepare the sample surface for analysis, the IM4000 Plus ion milling Hitachi machine was utilized, allowing for precise surface preparation and optimal imaging quality. Figure 2 provides a schematic representation of a prepared sample after the ion milling process. These techniques were crucial in obtaining accurate and reliable results for the analysis of the oxide layers.

## 3. Results

### 3.1. Influence of Oxygen Content on Discoloration

**Figure 3:**
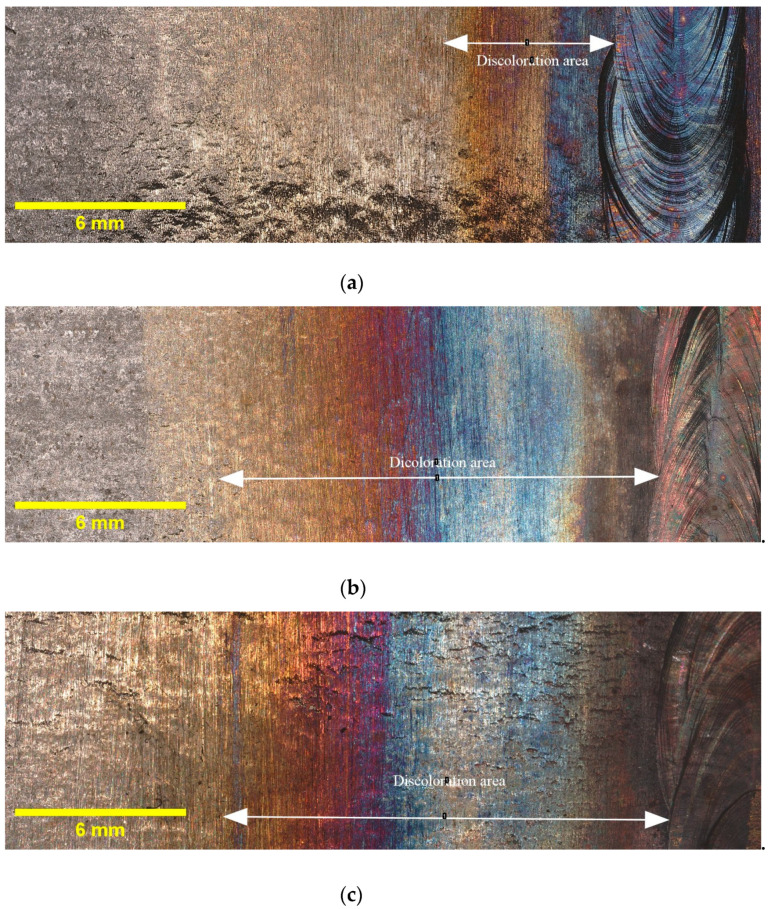

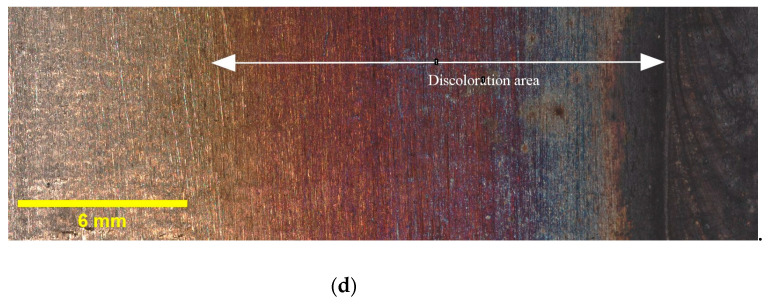
Discoloration zone with different oxygen concentrations in purging gas: (**a**) 50 ppm, (**b**) 200 ppm, (**c**) 500 ppm, and (**d**) 5000 ppm.

Figure 3 provides illustrative examples of the extent of discoloration that was observed in the HAZ of the tested samples with different oxygen concentrations. Additionally, it must be noted that the calculated widths reported in Table 4 are average values obtained from three samples taken from different locations along the circumference of the pipe. The results indicate that the highest discoloration width occurs for 5000 ppm oxygen, whereas the differences in width diminish as the oxygen content rises. Elevated oxygen levels can lead to higher oxidation rates, particularly at elevated temperatures. This means that the rate at which the metal reacts with oxygen to form oxides increases. Consequently, the discoloration width expands as more metal reacts, forming an oxide layer [25]. This finding suggests that the presence of oxygen plays a crucial role in the formation and extent of discoloration in the HAZ. These findings, which indicate variations in the discoloration width based on different oxygen contents, will be utilized in the subsequent sections of this paper to deepen our understanding of the relationship between oxygen content and discoloration, and its impact on pitting corrosion. As discussed in Section 1, the colors of the oxide layers correspond to different oxide thicknesses, and each welding condition exhibits a specific discoloration pattern. Therefore, it is crucial to determine the width of discoloration for each welding condition to gain comprehensive insights into the corrosion behavior.

### 3.2. Thermodynamic Calculation

**Figure 4:**
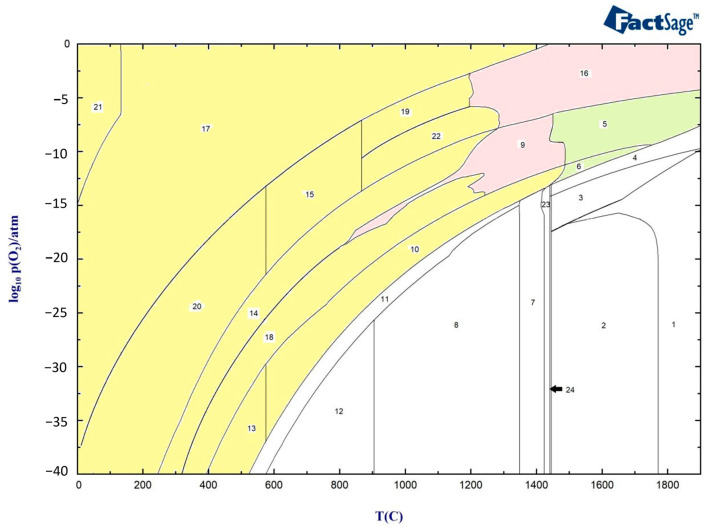
Oxide scale formation at different temperatures in 316L SS. Yellow zones: Oxide structure without Liquid oxide phase, Pink zones: Oxide structure with Oxide liquid phase, Green zones: Oxide structure with liquid phase and Oxide liquid phase.

Figure 4 presents the oxide scale formation at different temperatures in 316L SS, calculated using FactSage, and represents the different zones of the HAZ, ranging from the weld pool to the base metal. The type and structure at various temperatures and different partial pressures of oxygen indicate the amount of oxygen available from the air to the base material.

The above graph indicates that the oxide layer contains Spinel, Olivine, and Corundum with different concentrations until around 1300 °C, when delta ferrite begins to form (yellow zone). At around 1300 °C, the formation of oxide liquid begins to increase with the higher partial pressure of oxygen, as shown in the pink zones. Between 1423 °C and 1445 °C, the material experiences a mushy zone consisting of delta ferrite, austenite, and liquid metal, after which the base metal starts to melt at temperatures above 1445 °C.

**Figure 5:**
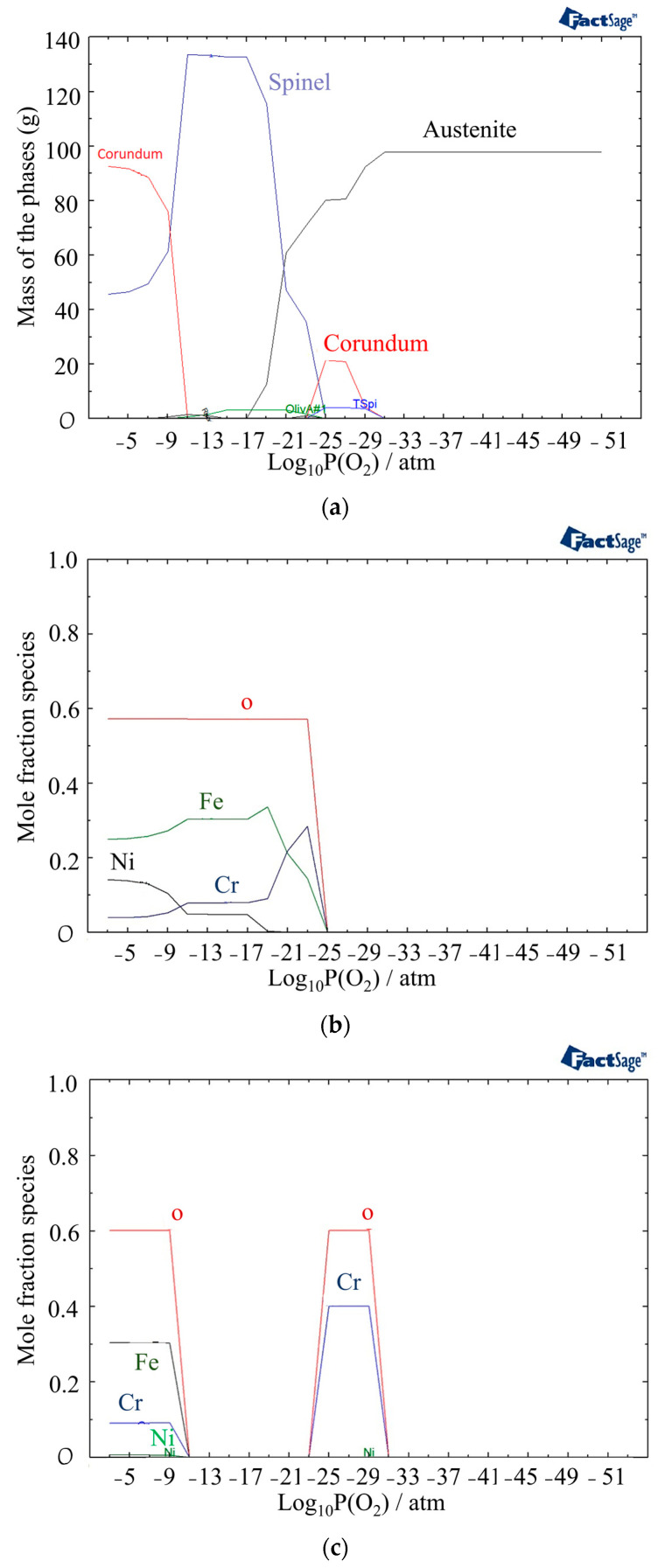
(**a**) Mass fractions of different phases at 800 °C as a function of partial pressure of oxygen, (**b**) Mole fraction of elements in the Spinel phase, (**c**) Mole fraction of elements in the Corundum phase.

In Figure 4, the mass fractions of different phases at 800 °C under different partial pressures of oxygen are shown using the Equilib module of FactSage software version 8.2 (Thermfact Ltd., Mount Royal, QC, Canada). The figure indicates the presence of the Corundum phase for two different ranges of partial pressures of oxygen. The first layer of Corundum, which contains chromium, nickel, and oxygen, is present between 10^−31^ and 10^−27^ atm, while the second layer, which is the outer layer of the oxide and begins at 10^−9^ atm, contains Cr, Fe, Ni, and oxygen (Figure 5c). The other phase observed after 10^−25^ atm is the Spinel phase consisting of Ni, Fe, Cr, and oxygen. In the Spinel phase, chromium is found to be more stable until around a 10^−12^ atm partial pressure of oxygen, followed by iron becoming more stable. Nickel is formed at 10^−15^ atm and becomes more stable than chromium after 10^−9^ atm (Figure 5b).

**Figure 6:**
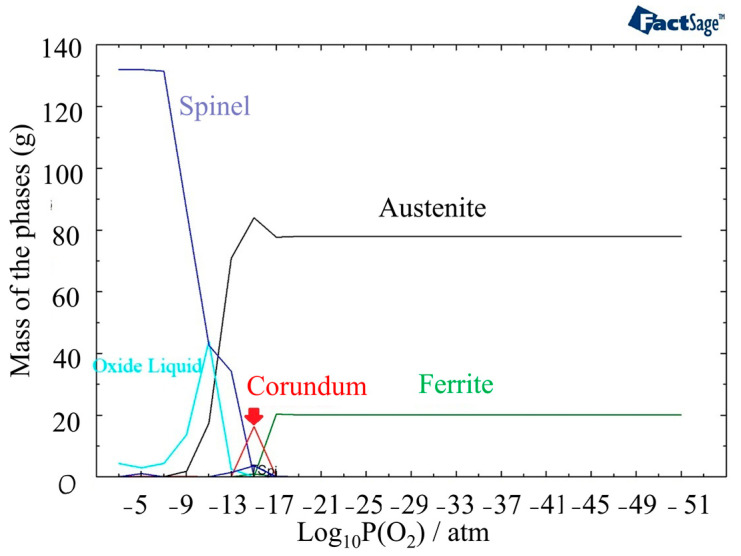
The structure of oxide formation at 1400 °C in 316L SS for various partial pressures of oxygen.

Oxide formation is initiated at a higher partial pressure of oxygen, specifically at a level of 10^−17^ atm, when the temperature reaches 1400 °C (Figure 6). This indicates that the oxide formation process is more favorable at higher temperatures compared to 800 °C. The amount of the Corundum phase becomes less and appears between 10^−17^ atm and 10^−13^ atm. Oxide liquid starts to form after a partial pressure of oxygen of 10^−16^ atm in the outer layer of oxide, but it is less stable than the Spinel phase.

**Figure 7:**
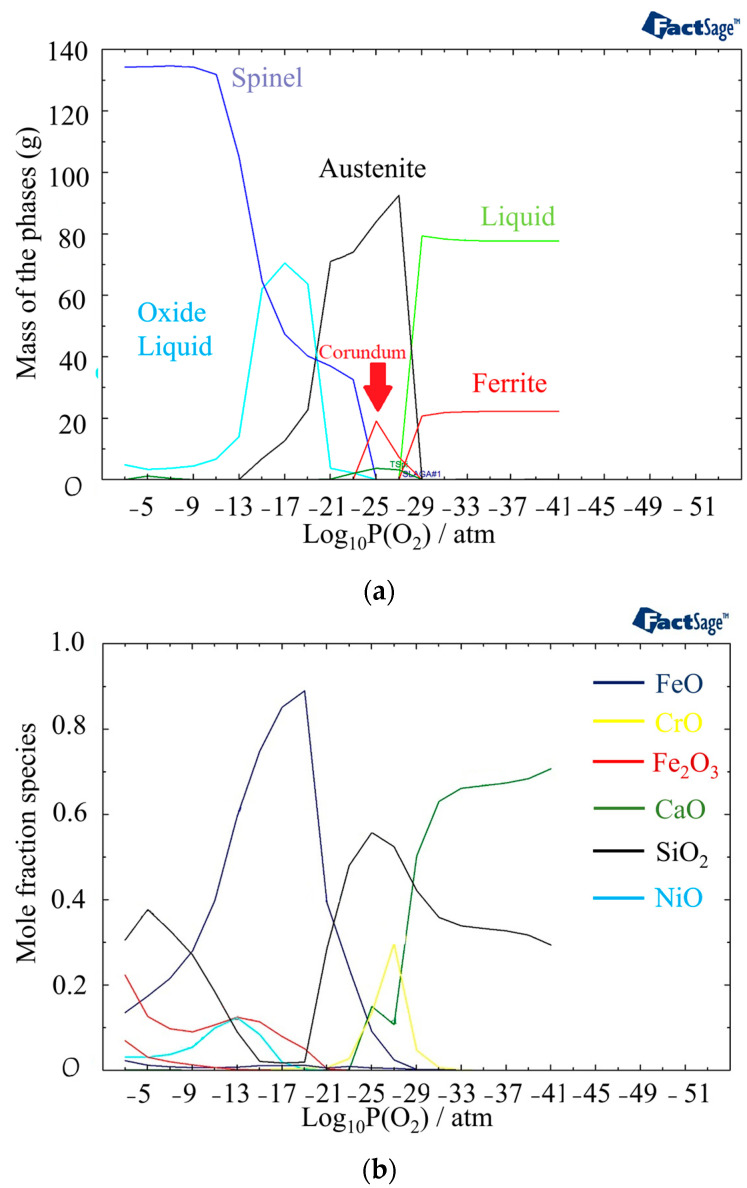
(**a**) Mass fraction of different phases at 1440 °C as a function of partial pressure of oxygen. (**b**) Component distribution in the oxide liquid phase.

In Figure 7a, the phases present at 1440 °C and different partial pressures of oxygen are displayed. Compared to 1400 °C, the outer layer contains a higher amount of oxide liquid. Figure 5b provides information on the phases present in the oxide liquid at different partial pressures of oxygen. The most stable phase between 10^−21^ atm to 10^−9^ atm is FeO. The inner and outer layers of the oxide liquid phase contain SiO_2_, a stable phase at these pressures. In addition, there are small amounts of Fe_2_O_3_ and NiO present in the outer layer and CrO in the inner layer. These analyses of the oxide composition and phase transitions provide valuable insights into the behavior of the material under different temperature and oxygen partial pressure conditions. Indeed, the presence of Spinel, Olivine, and Corundum phases, with varying proportions, indicates the complex nature of the oxide layer that is formed at the surface of the material. The identification of specific phases and their stability under different conditions allows for a better understanding of the structure of the phases in different discolored zones. The following sections delve into the impacts of specific phases on the corrosion resistance of the material.

### 3.3. Oxide Geometrical Characteristics

**Figure 8:**
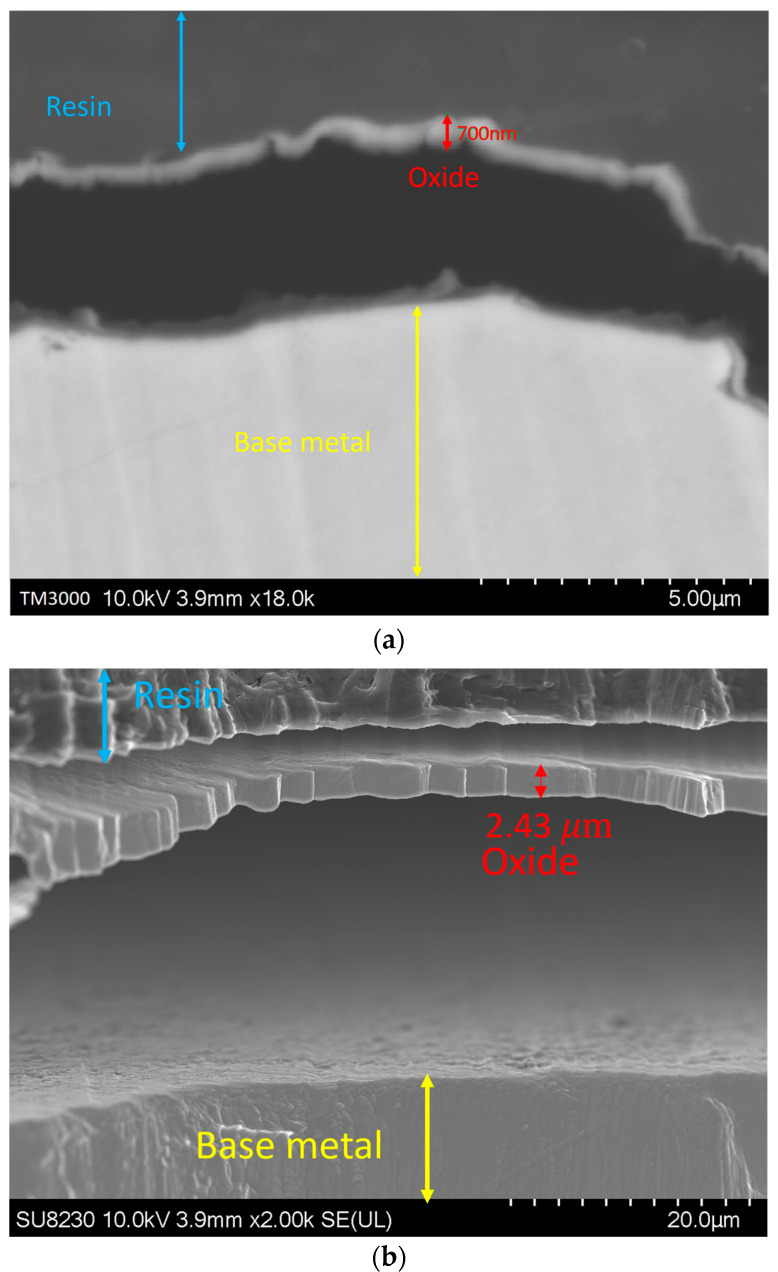

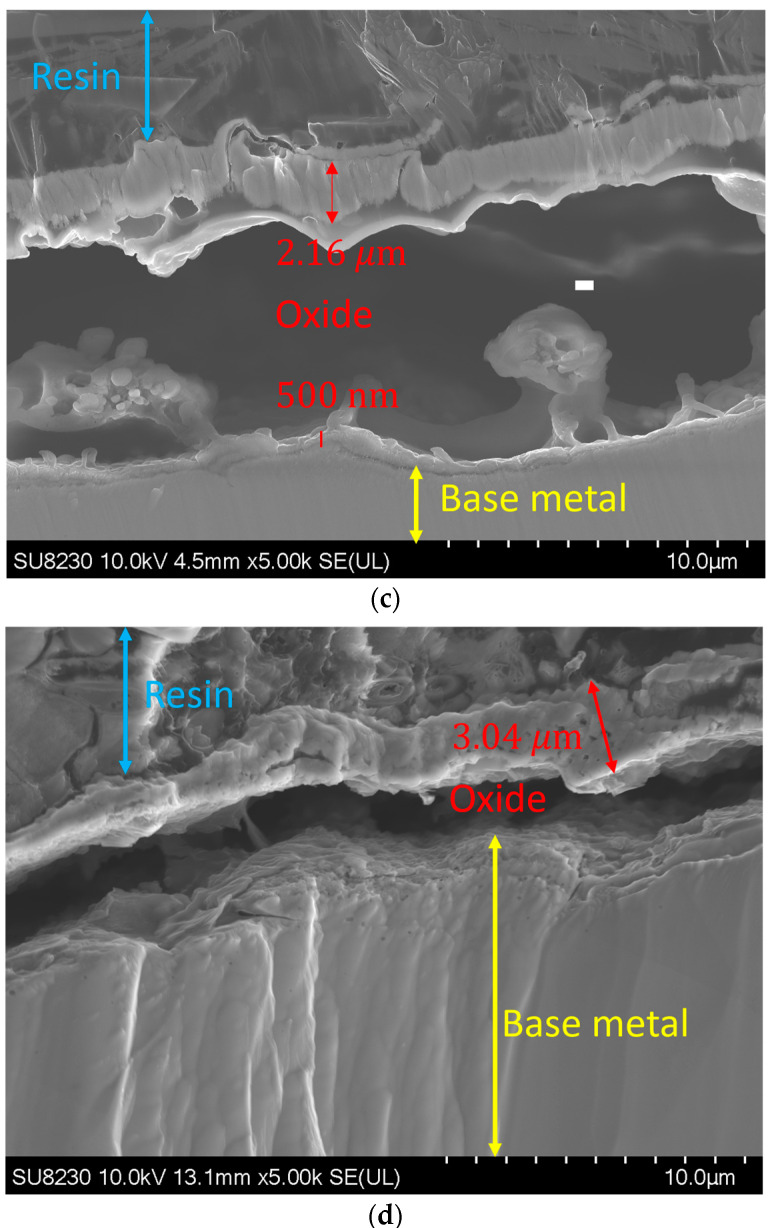
Oxide layer at a distance of 1.5 mm from the fusion line: (**a**) 50 ppm, (**b**) 200 ppm, (**c**) 500 ppm, and (**d**) 5000 ppm.

By increasing the oxygen content in the backing gas, the growth of the oxide layer was observed. Figure 8 illustrates such a variation in the oxide layer thickness with the oxygen content for a given use case. The image was taken at a distance of 1.5 ± 0.3 mm from the fusion line, where the thickness of the oxide layer increases from 700 ± 300 nm for 50 ppm oxygen to 2.7 ± 0.3 μm, 2.8 ± 0.3 μm, and 3 ± 0.3 μm for 200, 500, and 5000 ppm oxygen, respectively. It must be noted that other images were taken at other distances from the weld line, and they all showed the same trend.

In order to comprehensively analyze the kinetics of oxide growth in the weld zone of 316L SS, it is essential to consider the combined effects of oxygen content in the atmosphere and the temperature. To incorporate the impact of the oxygen content, we can introduce a term which represents the partial pressure of oxygen (*P*_*O*_2__). Assuming a power-law relationship between the rate constant and the partial pressure of oxygen, the equation becomes [26]:

(2) $$K=A\times {\left(P_{O_{2}}\right)}^{n}$$

In this equation, A is a constant and n denotes the power-law exponent that describes the dependence of the rate constant on the oxygen partial pressure.

Next, we can consider the Arrhenius equation, which relates the rate constant to temperature [27]:

(3) $$K=A^{\prime }\times e^{-\frac{E_{a}}{\mathrm{RT}}}$$

Using the two equations, we get:

(4) $$K\alpha \;{\left(P_{O_{2}}\right)}^{n},e^{-\frac{E_{a}}{\mathrm{RT}}}$$

In this equation, assuming a linear relationship between the oxide thickness and time (Wagner equation), X = Kt:

(5) $$X\alpha \;{\left(P_{O_{2}}\right)}^{n},e^{-\frac{E_{a}}{\mathrm{RT}}}$$

Where t is the time and X is the oxide thickness. This equation shows that the oxide thickness also increases with the oxygen content and temperature.

**Figure 9:**
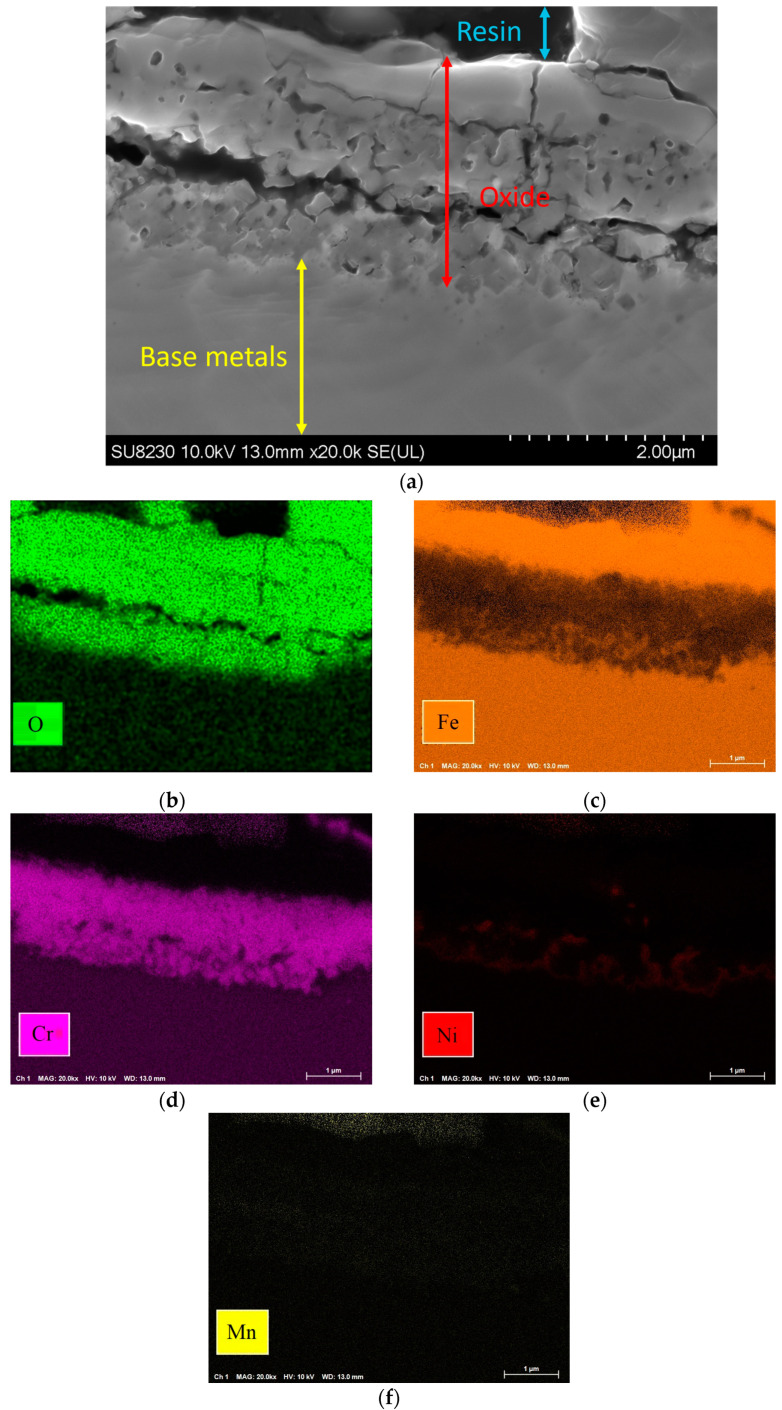
(**a**) Oxide scale at 1.7 mm distance from fusion line of 5000 ppm oxygen content and elemental map: (**b**) O, (**c**) Fe, (**d**) Cr, (**e**) Ni, (**f**) Mn.

**Figure 10:**
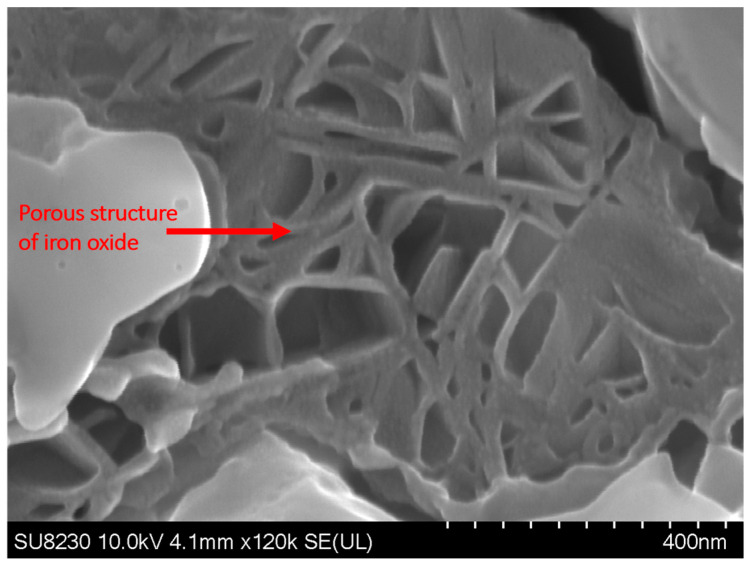
SEM image of the surface of the sample with 5000 ppm oxygen content, showing the presence of porous structures near the fusion line.

Figure 9 shows the elemental map of the oxide layer at a distance of 1.7 mm from the fusion zone in the sample with 5000 ppm oxygen content. The image reveals that the inner layer contains iron, Cr, and Mn, which are Spinel and Corundum phases based on Figure 5, Figure 6 and Figure 7. The outer layer consists of iron oxide, which is a mixture of the outer Spinel with an iron-rich area, and the second layer of Corundum. Moreover, there is a porous structure that appears in the 5000 ppm sample near the fusion line, possibly an oxide liquid with an iron oxide structure [28,29]. These porous structures are shown in Figure 10. The findings presented in this section highlight the influence of oxygen content on the oxide layer’s thickness, composition, and morphology. This knowledge is crucial to understanding pitting corrosion behavior. The quantitative data and visual evidence provided in the figures above serve as a foundation for further analysis and discussions in the subsequent section of the paper.

### 3.4. Pitting Corrosion Tests

**Figure 11:**
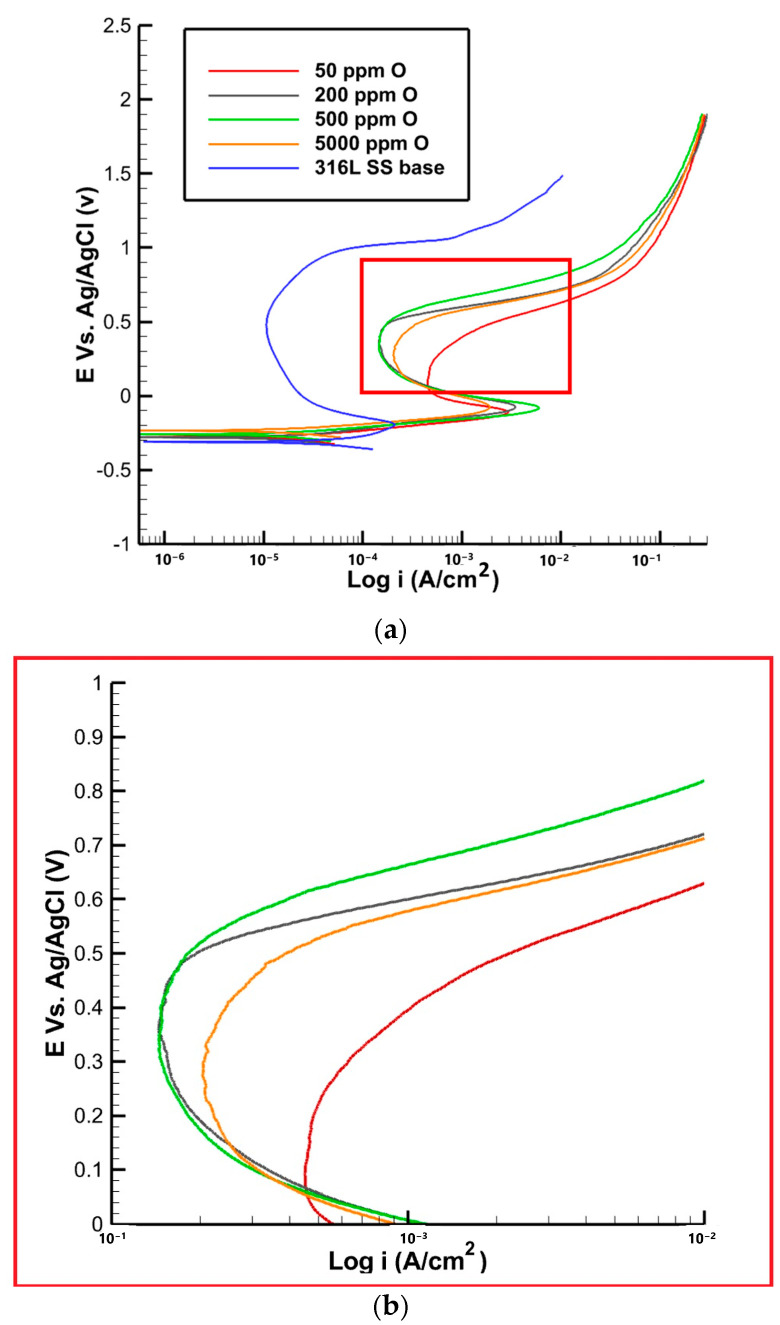
(**a**) Polarization curve of 316L SS with varying oxygen content, (**b**) high magnification of the red area in the plot (**a**).

The polarization curves and corrosion parameters for the samples with different oxygen contents are presented in Table 5 and Figure 11. As shown in Table 5, the passive current densities for all samples initially increased with the increase in oxygen content up to 500 ppm, followed by a decrease in the sample with 5000 ppm. This can be attributed to the porous structure observed in the oxide layer near the fusion line of the sample with 5000 ppm, which may have led to the formation of more active sites for corrosion. For the sample with 50 ppm of oxygen, the passive current was determined to be 463 ± 10 µA. This value indicates the rate of spontaneous oxidation processes occurring on the surface, suggesting a moderate level of corrosion resistance. As the oxygen content increased to 200 ppm, the passive current decreased to 210 ± 10 µA. This reduction in passive current density suggests an improvement in the corrosion resistance of the material, as a more protective passive film formed on the surface. Further increasing the oxygen content to 500 ppm led to a slightly higher passive current of 230 ± 20 µA. Although the passive current density increased slightly, it still indicates a relatively good corrosion resistance compared to the sample with 50 ppm. However, in the sample with 5000 ppm of oxygen, the passive current exhibited a further increase to 290 ± 30 µA. This unexpected rise in the passive current density suggests a slight decrease in the corrosion resistance compared to the samples with lower oxygen contents. The presence of a porous oxide structure near the fusion line, as observed in the composition analysis, contributed to this phenomenon by creating more active sites for corrosion [30].

In terms of the pit current densities, as shown in Figure 11, the pit current was measured to be 463 ± 10 µA. This can be attributed to the limited availability of oxygen, which impedes the formation of a fully protective passive film. The lower oxygen content restricts the extent of passivation, rendering the material more susceptible to localized corrosion. As the oxygen content increased to 200 ppm, the pit current decreased to 200 ± 10 µA. Further increasing the oxygen content to 500 ppm resulted in a slightly higher pit current of 210 ± 30 µA. This can be attributed to the formation of a more stable and protective passive film on the material’s surface. The presence of oxygen promotes the development of this film, enhancing the material’s resistance to localized corrosion. Interestingly, for samples with 5000 ppm of oxygen, the pit current density did not decrease as anticipated but remained slightly elevated. This behavior can be attributed to the presence of a porous oxide structure near the fusion line. The porous nature of the oxide layer creates preferential sites for localized corrosion initiation, contributing to the higher pit current density.

The passive potential values are reported in Table 5, showing zero for the sample with 50 ppm oxygen, indicating the absence of a stable passive film. In contrast, the passive potential value increases to 0.05 V for the sample with 200 ppm oxygen and then to 0.07 V and 0.09 V for the two other samples with 500 and 5000 ppm oxygen, respectively. The obtained results indicate that, by increasing the oxygen content, the formation of the passive film is accelerated. In the base metal, where there are no oxides present, the passive potential is lower when compared to the samples with discoloration. At 50 ppm of oxygen, the pitting potential was found to be 0.42 V. This relatively higher pit potential suggests a greater susceptibility to localized corrosion. The pitting potential of the base metal was significantly higher compared to the sample with discoloration, which measures at 0.96 V. This disparity can be attributed to the heat and temperature experienced during welding in the heat-affected zone. The limited availability of oxygen at this concentration may hinder the formation of a robust passive film, making the material more vulnerable to localized attack. As the oxygen concentration increased to 200 and 500 ppm, the pit potential increased to 0.53 and 0.54 V, respectively. This indicates a slight improvement in the resistance to localized corrosion. The higher pit potentials suggest the formation of a more stable and protective passive film, which provides better defense against localized corrosion initiation and propagation. In samples with 5000 ppm of oxygen, the pitting potential decreased to 0.49 V. This decrease in pit potential can be attributed to the presence of a porous iron oxide structure near the fusion line, as observed in previous analyses. The porous nature of the oxide layer promotes the formation of localized corrosion sites, resulting in a reduction in the pit potential. This implies a higher susceptibility to localized corrosion, potentially compromising the integrity of the material.

## 4. Conclusions

This research study presents an investigation into the corrosion behavior and the influence of oxygen content on the properties of a welded 316L SS pipeline in the root joint area. By exploring the microstructural characteristics, polarization curves, and corrosion parameters of the studied material, valuable insights are gained into the material’s corrosion resistance. The investigation presented in this work yields the following results:

1. The width of discoloration increased with increasing oxygen content, and notable differences in color were observed between the samples with 5000 ppm and 500 ppm/200 ppm of oxygen. In particular, the 5000 ppm sample exhibited an opaque zone located behind the welding line, indicating distinct characteristics in the oxide formation process at high oxygen concentrations.
2. The results indicate that the oxide thickness increases with an increase in the oxygen content of the purging gas, when it ranges from 50 to 5000 ppm. This suggests that a higher concentration of oxygen promotes the formation of a thicker oxide layer on the surface of the material. The observed trend in oxide thickness provides valuable insights into the influence of oxygen content on the oxide formation process and its subsequent impact on the corrosion resistance of the material.
3. The oxide structure was found to be a mixture of Spinel and Corundum phases, with the presence of an oxide liquid phase at high temperatures. The oxide liquid phase exhibited a higher concentration of FeO, which resulted in a porous structure.
4. The pitting corrosion resistance showed an increasing trend up to a 500 ppm oxygen content, followed by a decrease at 5000 ppm. The sample with 500 ppm exhibited the highest pit potential, suggesting the formation of a more stable passive film that is less susceptible to localized corrosion. The presence of a porous oxide structure near the fusion line in the 5000 ppm sample may have contributed to its lower pitting corrosion resistance.

## Figures and Tables

**Table 1:** Chemical composition of 316L SS (wt %).

**Element**	**C**	**Mn**	**Si**	**P**	**S**	**Mo**	**Cr**
Amount	0.02	0.97	0.43	0.28	0.001	2.05	16.47
**Element**	**Ni**	**Al**	**Co**	**Cu**	**W**	**N**	**Other**
Amount	11.37	0.0162	0.22	0.25	0.073	0.093	0.13

**Table 2:** Welding condition parameters of 316L SS samples.

Process	GTAW	GTAW	GTAW	GTAW
**Pass**	1	2	3	4
**Shielding gas**	Ar	Ar	Ar	Ar
**Purge gas flow rate (CFH)**	40	40	40	40
**Current (A)**	125–202	166–215	200–223	208–243
**Voltage (V)**	10.6–14	11.8–15.7	12.8–16.4	13.8–16.4
**Tube to work distance (mm)**	9.52	9.52	9.52	9.52
**Heat In Put (kJ.mm^−1^)**	1.31	1.17	1.28	1.44
**Filer material**	ER316L (Exocor)	ER316L (Exocor)	ER316L (Exocor)	ER316L (Exocor)

**Table 3:** Purging gas concentrations.

Oxygen Content (PPM)	50	200	500	5000
Component	Ar + 0.005% O	Ar + 0.02% O	Ar + 0.05% O	Ar + 0.5% O

**Table 4:** Components in different zones.

Zone	Phase
1	Liquid
2	Liquid + MeS
3	Liquid + alfa Ca_2_SiO_4_
4	Liquid + Oxide Liquid
5	Liquid + Oxide Liquid + Spinel
6	Liquid + Oxide Liquid + Corundum + SiO_2_ + TSpinel
7	Delta ferrite + Austenite + MeS
8	Austenite + MeS
9	Oxide Liquid + Austenite + Spinel + TSpinel
10	Austenite + MeS + Corundum + SiO_2_ + TSpinel + Ca_3_Cr2Si_3_O_12_
11	Austenite + MeS + Wollastonite
12	Austenite + MeS
13	Austenite + MeS + Corundum + SiO_2_ + TSpinel + Wollastonite
14	Spinel + Austenite + Olivine + Beta Ni_3_S_2_
15	Spinel + Olivine + Rhodonite + Pyrrhotite
16	Oxide Liquid + Spinel
17	Spinel + Corundum + SiO_2_ + MnSO_4_ + CaSO_4_
18	Austenite + MeS + Spinel + Rhodonite + TSpinel + Wollastonite
19	Spinel + Olivine + Rhodonite + MnSO_4_ + CaSO_4_
20	Spinel + Corundum + Olivine + CaSO_4_ + MnSO_4_
21	Spinel + Corundum + SiO_2_ + MnSO_4_ + CaSO_4_ + MnO_2_ + CrO_3_
22	Spinel + Olivine
23	Delta ferrite + Austenite + MeS + Liquid
24	Delta ferrite + MeS + Liquid

**Table 5:** Corrosion parameters of 316L SS with varying oxygen content.

Oxygen Content (PPM)	50	200	500	5000	316L SS Base
i_passive_ (µA)	463 ± 10	210 ± 10	230 ± 20	290 ± 30	198 ± 30
i_pit_ (µA)	463 ± 10	200 ± 10	210 ± 10	260 ± 20	297 ± 30
E_passive_ (V)	0 ± 0.01	0.05 ± 0.03	0.07 ± 0.03	0.09 ± 0.01	−0.04 ± 0.01
E_pit_ (V)	0.42 ± 0.06	0.53 ± 0.01	0.54 ± 0.01	0.49 ± 0.02	0.96 ± 0.01
